# Risk Assessment of Roundabout Scenarios in Virtual Testing Based on an Improved Driving Safety Field

**DOI:** 10.3390/s24175539

**Published:** 2024-08-27

**Authors:** Wentao Chen, Aoxue Li, Haobin Jiang

**Affiliations:** 1 Automotive Engineering Research Institute, Jiangsu University, Zhenjiang 212013, China; takumii@foxmail.com (W.C.); jianghb@ujs.edu.cn (H.J.); 2 School of Automotive and Traffic Engineering, Jiangsu University, Zhenjiang 212013, China

**Keywords:** autonomous vehicles, scenario-based testing, roundabout risk assessment, driving safety field

## Abstract

With the advancement of autonomous driving technology, scenario-based testing has become the mainstream testing method for intelligent vehicles. However, traditional risk indicators often fail in roundabout scenarios and cannot accurately define dangerous situations. To accurately quantify driving risks in roundabout scenarios, an improved driving safety field model is proposed in this paper. First, considering the unique traffic flow characteristics of roundabouts, the dynamic characteristics of vehicles during diverging or merging were taken into account, and the driving safety field model was improved to accurately quantify the driving risks in roundabout scenarios. Second, based on data from the rounD dataset, the model parameters were calibrated using the social force model. Finally, a DENCLUE-like method was used to extract collision systems, calculate vehicle risk degree, and analyze these risks for both the temporal and the spatial dimensions, providing guidance for virtual testing. The proposed method significantly improves detection efficiency, increasing the number of identified dangerous scenarios by 175% compared to the Time to Collision (TTC) method. Moreover, this method can more accurately quantify driving risks in roundabout scenarios and enhance the efficiency of generating dangerous scenarios, contributing to promoting the safety of autonomous vehicles.

## 1. Introduction

Despite continuous advancements in autonomous driving technology, the frequent occurrence of traffic accidents, particularly in complex traffic environments [1], underscores that traffic safety remains a pressing concern and that autonomous driving still presents significant safety risks at this stage. To ensure the safety of autonomous vehicles, it is essential to evaluate their safety performance through comprehensive testing methods, alongside the development of autonomous vehicle features. Traditional public road testing is slow, costly, and inefficient, failing to meet the needs of high-level autonomous vehicle testing. In response, scenario-based testing has emerged as a preferred approach, offering targeted and comprehensive validation in perception, decision-making, and vehicle control [2] by effectively simulating complex traffic scenarios to assess the stability and safety of autonomous driving systems under varying conditions [3]. Safety-critical scenarios are crucial for diagnosing functional defects in autonomous vehicles. Acquiring these random scenarios directly from natural driving data is highly inefficient [4]. Therefore, identifying and discovering safety-critical scenarios is a complex and challenging task.

To effectively assess the safety of autonomous vehicles, methods for generating safety-critical scenarios have garnered increasing attention. NHTSA has summarized test scenarios for autonomous driving systems [5] and provided a detailed simulation framework, scenario description standards, and regulatory standards for testing high-level automated driving systems [6]. Meanwhile, scholars have utilized various techniques to generate safety-critical scenarios, including resampling [7] by extracting the distributions of key variables (e.g., relative distance), as well as adversarial generation [8] and rule-based approaches [9]. Ding et al. argue that the most important factor in generating safety-critical scenarios is selecting the key variables to quantify driving risk [10]. Correct risk quantification helps an autonomous driving system identify truly challenging, test-worthy scenarios, whereas incorrect risk quantification produces useless scenarios. These useless scenarios are either too trivial for the system or too rare in the real world. Traditional metrics such as Time to Collision (TTC), Time Headway (THW), and Proportion of Stopping Distance (PSD) can reflect the danger in autonomous driving scenarios to some extent, but their individual limitations prevent a comprehensive and accurate measurement of driving risk. Therefore, developing more comprehensive and effective danger metrics has become a key issue in current research.

Existing metrics for assessing driving risk include time/distance-based metrics, data-based metrics, and field-based metrics [11]. Time/distance-based metrics are the most commonly used methods for assessing driving risk. For example, Time to Collision (TTC) and Time Headway (THW) are widely recognized [12]. These methods effectively quantify risk by analyzing the velocity and distance relationships between vehicles. Some scholars believe that using only Time to Collision (TTC) is insufficient to describe the risks during driving. Therefore, they have developed Time-Exposed Time-to-Collision (TET) and Time-Integrated Time-to-Collision (TIT) [13]. Liu et al. [14] proposed the Conflict Severity Index (CSI) in roundabout scenarios, which integrates TTC and MaxDeltaV to indicate the potential severity of collisions and uses TTC to indicate the likelihood of collisions. The above metrics are widely used and offer better real-time performance. However, they mainly define conflicts between two vehicles. In roundabout scenarios, due to complex traffic flow and multi-directional intersections [15], multi-vehicle conflicts are very common, especially at multi-lane roundabouts [16]. Therefore, time/distance-based metrics have limitations in roundabout scenarios and fail to accurately assess driving risk.

With the rapid advancement of machine learning, data-based metrics have become crucial tools in vehicle risk assessment. Katrakazas et al. [17] developed a novel risk assessment method that integrates network-level crash estimation with risk assessment in real-time, using an interactive perceptual motion modeling framework and Dynamic Bayesian Networks (DBNs). Chen et al. [18] used the Deep Q-Network (DQN) algorithm to combine reinforcement learning with a safety monitoring mechanism, optimizing intelligent lane-changing decisions and risk assessment for vehicles. Joo et al. [19] used a Multivariate Bayesian Structural Time Series (MBSTS) model to predict the future actions of adjacent vehicles and assessed the risk of lane changes for Connected Autonomous Vehicles (CAVs) through virtual lane change scenarios and reliability analysis methods. However, data-based metrics fail to adequately consider the complex interactions and frequent merging and diverging behaviors in roundabout scenarios, and their accuracy depends on the quality of the scenarios and data, with poor model interpretability.

Field-based metrics, which account for multi-vehicle interactions, provide a promising solution for quantifying risk in roundabout scenarios. This approach provides a more comprehensive understanding of vehicle behavior in complex traffic environments by modeling the interaction between vehicles. In the early days, field-based methods were commonly used for path planning and vehicle control [20,21]. In 2015, Wang et al. [22] analyzed the influencing factors of driving risk and proposed a generic model of the driving safety field that considers the interaction of human–vehicle–road factors. This model overcomes the shortcomings of traditional methods in dealing with complex traffic situations and defines a risk quantification index, the Driving Safety Index (DSI), based on the driving safety field. Zheng et al. [23] constructed a safety field model through a new framework, viewing vehicle motion on the road as a force field and using a Hidden Markov Model (HMM) to predict the driver’s steering intentions. Li et al. [24] proposed a new potential field model based on the driving safety field, incorporating the effects of acceleration and heading angle on driving risk. The potential field dynamically adjusts with the vehicle’s movement to more accurately reflect the actual driving risks in various motion states. Ma et al. [25] improved the driving safety field to accurately assess the driving risks in the weaving areas of highways by considering the specific driving characteristics of these areas. Han et al. [26] proposed a novel Spatio-Temporal Risk Field (STRF), which differs from existing studies, which only obtain instantaneous safety fields by characterizing dynamic driving risk from a spatio-temporal coupling perspective.

However, the existing driving safety field models fail to accurately capture the unique driving characteristics of roundabout scenarios, leading to limitations in their applicability and precision. To overcome these challenges, this study aims to develop an improved driving safety field model specifically designed to model driving risks in roundabouts, addressing the gaps in applicability and accuracy of existing models.

The key contributions of this work are twofold. First, by utilizing the concept of the driving safety field, this study introduces a DENCLUE-like method to calculate the danger degree in roundabout scenarios. Second, the model further analyzes the risk degree for both temporal and spatial dimensions, enabling a more comprehensive and accurate assessment of scenario risks.

**Figure 1:**
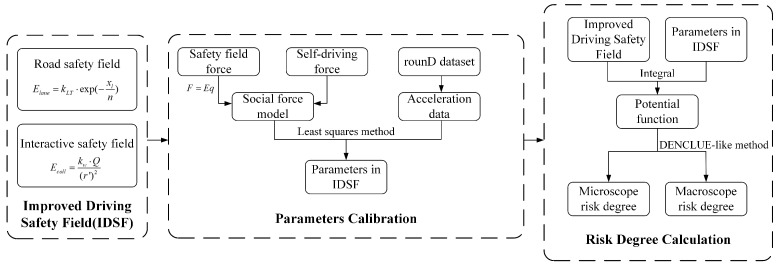
Framework of the proposed model.

Figure 1 illustrates the overall framework of this paper.

## 2. Improved Driving Safety Field Model

In physics, the concept of a field refers to a distributed entity endowed with a physical quantity at every point in space. The concept of a driving safety field draws on this theory and aims to systematically describe and analyze the spatial and temporal distribution properties of various risk factors during driving. The driving safety field is a dynamic risk field influenced by objective factors such as road conditions, traffic flow, and weather. In the driving safety field, the intensity and distribution of risk change with time and the external environment, thus affecting the driver’s decision-making and behavior.

The improved driving safety field consists of the road safety field and the interactive safety field.

### 2.1. Road Safety Field

**Figure 2:**
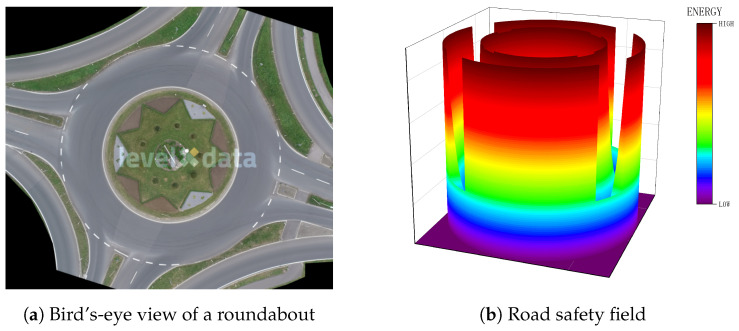
Sketches of the road safety field.

The road safety field refers to the restraining effect of lane markings, which help keep a vehicle centered within its lane. Lane markings are categorized into two types: those that allow lane changes (e.g., white dashed lines) and those that restrict them (e.g., solid boundary lines). Sketches of the road safety field are shown in Figure 2.

**Figure 3:**
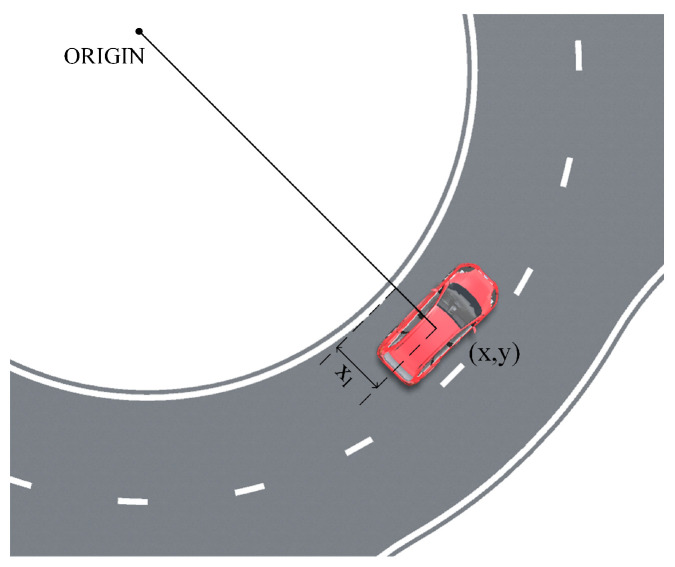
Calculation diagram of $x_{l}$.

Existing road safety fields are mostly designed for straight roads in urban areas or highways. Considering the unique structure and one-way traffic flow of roundabouts, there are no double yellow lines (which separate opposing traffic flows) within roundabouts. Instead, only lane centerlines and road boundary lines are present. The road safety field is defined by the following equation:

(1) $$E_{lane}=k_{LT}\cdot \exp\left(-\frac{|x_{l}|}{n}\right)\cdot \frac{x_{l}}{|x_{l}|},$$

where $k_{LT}$ is the lane type coefficient; $x_{l}$ represents the vehicle’s coordinate relative to the lane line (calculated as shown in Figure 3); and *n* is the shape coefficient, which determines the rate of change of the field strength.

### 2.2. Interactive Safety Field

The interactive safety field represents the impact of static or dynamic traffic participants on driving safety. Drivers typically maintain a safety buffer around the vehicle during movement, keeping a distance from nearby risks to ensure an acceptable level of collision risk. This safety buffer zone represents the inherent safety field generated by the moving vehicle, similar to how a charged particle emits an electric field.

**Figure 4:**
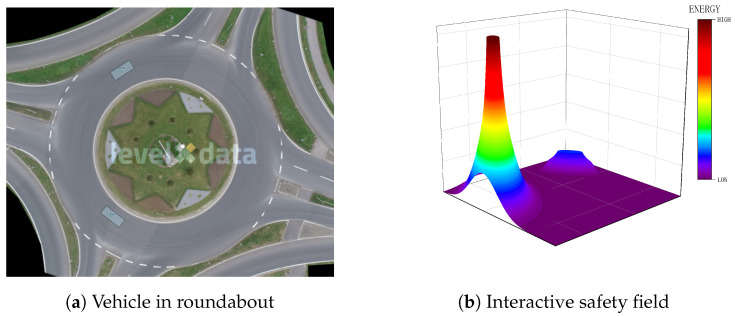
Sketches of the interactive safety field.

The concept of an interactive safety field is analogous to an energy field in physics. Therefore, the safety field theory, as applied to dynamic and static road users, draws on the electric field equation in its formulation [27]. The electric field equation considers three key variables: particle charge, distance from the particle measuring the field, and the medium’s effect. This makes the electric field equation suitable for incorporating risks due to the road user, the driving environment, and the distance from the road user. Sketches of the interactive safety field are shown in Figure 4.

In the roundabout scenario, the vehicle’s heading angle changes continuously and dynamically. The distribution of the field must reflect and adapt to this continuously changing characteristic to correctly assess the risk. The interactive safety field can update in real time with changes in the heading angle using the coordinate transformation formula:

(2) $$\left[\begin{array}{c} x^{*}\\ y^{*} \end{array}\right]=\left[\begin{array}{cc} cos\varphi_{e} & sin\varphi_{e}\\ -sin\varphi_{e} & cos\varphi_{e} \end{array}\right]\left[\begin{array}{c} x\\ y \end{array}\right],$$

where (x,y) are the coordinates before the transformation; $\left(x^{*},y^{*}\right)$ are the coordinates after the transformation; $\varphi_{e}$ is the heading angle of the host vehicle.

In the existing safety field research, it is assumed that the risk of the host vehicle approaching an obstacle vehicle is the same from all directions [25,28]. However, in actual driving, the longitudinal speed of the vehicle is much greater than the lateral speed. Therefore, the pseudo-distance $d^{\prime }$ is used to appropriately amplify the risk in the longitudinal direction [29]:

(3) $$|d^{\prime }|=\sqrt{\delta_{1}{\left(\frac{x_{e}^{*}-x_{o}^{*}}{l_{e}}\right)}^{2}+\delta_{2}{\left(\frac{y_{e}^{*}-y_{o}^{*}}{w_{e}}\right)}^{2}},$$

where $\left(x_{e}^{*},y_{e}^{*}\right)$ and $\left(x_{o}^{*},y_{o}^{*}\right)$ are the transformed coordinates of the host vehicle and the obstacle vehicle, respectively; $\delta_{1},\delta_{2}$ are the ellipse shape control coefficients; $l_{e}$ is the length of the host vehicle; $w_{e}$ is the width of the host vehicle.

The potential risk *Q* represents the potential risk of a traffic participant to other individuals in the environment. According to Arun et al. [27], the potential risk *Q* varies with driver and vehicle characteristics, such as reaction time and maximum braking capability. However, obtaining these data in practice is challenging, leading to certain difficulties in practical applications.

**Figure 5:**
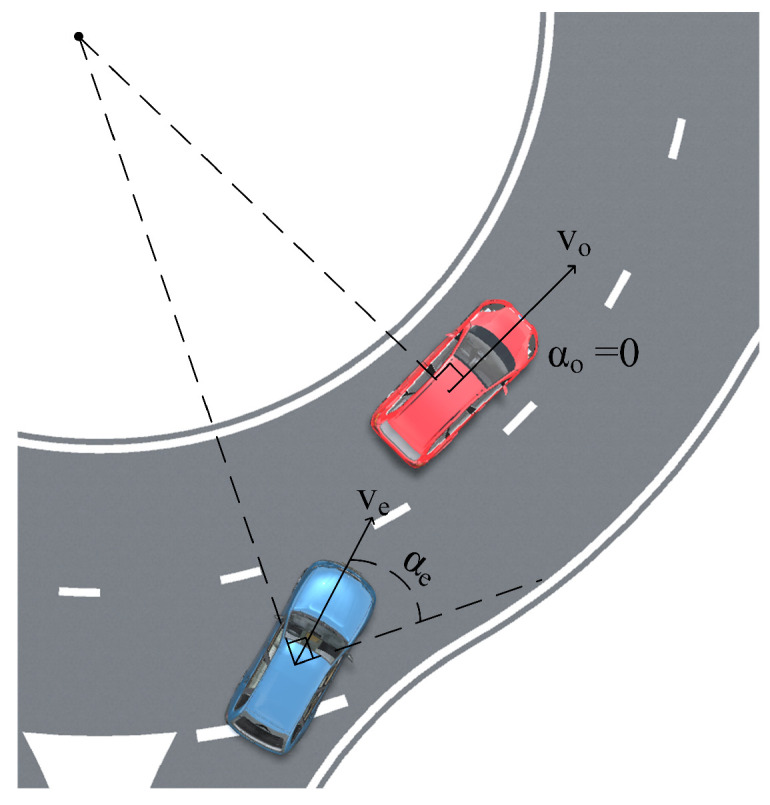
Calculation diagram of heading deviation.

In the context of autonomous vehicles, communication technology enables information exchange between vehicles and the road. This allows for the easy acquisition of kinematic parameters of other vehicles, such as speed, acceleration, and heading angle. Therefore, the potential risk of moving objects *Q* can be expressed by the following equation:

(4) $$Q=\left(k_{\alpha }\cdot \left|\alpha_{e}\right|+k_{v}\cdot v_{e}+k_{se}*l_{e}*w_{e}\right)\cdot T_{e},$$

where $\alpha_{e}$ is the heading deviation, used to describe the vehicle’s merging and diverging behavior in the roundabout, whose calculation method is shown in Figure 5; $v_{e}$ is the vehicle speed; $k_{\alpha },k_{v},k_{se}$ are the parameters to be determined; and $T_{e}$ is the vehicle type coefficient.

The interactive safety field is given by

(5) $$E_{coll}=\frac{k_{w}\cdot Q}{{\left(|d^{\prime }|\right)}^{2}}\cdot \frac{d^{\prime }}{|d^{\prime }|}$$

where $k_{w}$ is the weather coefficient; *Q* is the potential risk the object carries; and $d^{\prime }$ is the pseudo-distance.

Combining the known conditions, we can obtain

(6) $$E_{coll}=\frac{k_{w}\cdot \left[\left(k_{\alpha }\cdot \left|\alpha_{e}\right|+k_{v}\cdot v_{e}+k_{se}*l_{e}*w_{e}\right)\cdot T_{e}\right]}{{\left\{\sqrt{\delta_{1}{\left(\frac{x_{e}^{*}-x_{o}^{*}}{l_{e}}\right)}^{2}+\delta_{2}{\left(\frac{y_{e}^{*}-y_{o}^{*}}{w_{e}}\right)}^{2}}\right\}}^{2}}\cdot \frac{d^{\prime }}{|d^{\prime }|}.$$

In current studies on driving safety fields, the accuracy and applicability of the model largely depend on the proper configuration of parameters [28,30]. These parameters are used to adjust the weight of different influencing factors, reflecting the significance of each factor in various driving scenarios. Specifically, parameter tuning allows for precise control over factors such as heading deviation and speed variations within the model, thereby ensuring the model’s reliability and practicality in risk assessment. Therefore, the careful selection and calibration of these parameters are critical steps in driving safety field modeling.

### 2.3. Coefficient Determination

In the actual driving process, obtaining the vehicle’s mass is challenging. Therefore, this paper defines a substitute index that uses the projected area of obstacles or vehicles on the road to indirectly represent the mass [31], as shown in the following equation:

(7) m=T·l·w,

where *T* is the type coefficient; *l* is the length of the vehicle or obstacle; and *w* is the width of the obstacle. The type coefficient calibration results are shown in Table 1.

In risk degree calculations, the weather coefficient adjusts for the changes in risk degree due to varying weather conditions. Different weather conditions significantly impact the driving environment, so incorporating the weather coefficient in risk calculations allows for a more accurate assessment of traffic safety under current conditions.

The weather coefficient values are derived from the study by Malin et al. [32], as shown in Table 2.

According to the study by Li et al. [24], the coefficient values for the road potential field are shown in Table 3.

## 3. Risk Assessment with the DENCLUE-like Method

### 3.1. Potential Function

The potential function represents the potential energy at a specific point in the field. As a scalar, it represents the energy an object has due to its position in a force field, where higher potential energy corresponds to a higher risk state for the vehicle. The field strength function describes the distribution of force, but in driving risk analysis, focusing directly on the energy distribution through the potential function [33] can more effectively quantify risk. Additionally, since field strength is a vector and requires the consideration of direction in analysis, using the potential function simplifies this process.

In electric field theory, the potential function is obtained by integrating the electric field strength:

(8) $$V=-\int \vec{E}\cdot d\vec{l}.$$

Substituting into the field strength formula, we obtain

(9) $$\begin{array}{cc} V_{coll} & =\frac{k_{w}\cdot Q}{d^{\prime }},\\ V_{lane} & =n\cdot k_{LT}\cdot \exp\left(-\frac{x_{l}}{n}\right). \end{array}$$

### 3.2. DENCLUE-like Method

DENCLUE (Density-Based Clustering) is a clustering algorithm based on the density distribution in the data space [34]. The algorithm first constructs a density function by summing influence functions, then identifies cluster centers by searching for local maxima of the density function. Starting from each data point, the algorithm follows the density gradient to locate the local maximum, assigning the point to the corresponding cluster center. Finally, the algorithm considers the distance and density differences between cluster centers, merging them when they are sufficiently close with minimal density variation, producing the final clustering result.

In the roundabout scenario, each vehicle can be likened to a point charge with a surrounding safety field. Any other vehicle within this safety field will be affected, and this influence is additive. The danger distribution of a single vehicle is described by a potential function, which represents the influence of each vehicle on its surroundings in the roundabout scenario (influence function). The potential function is a scalar function and can be directly superimposed.

**Figure 6:**
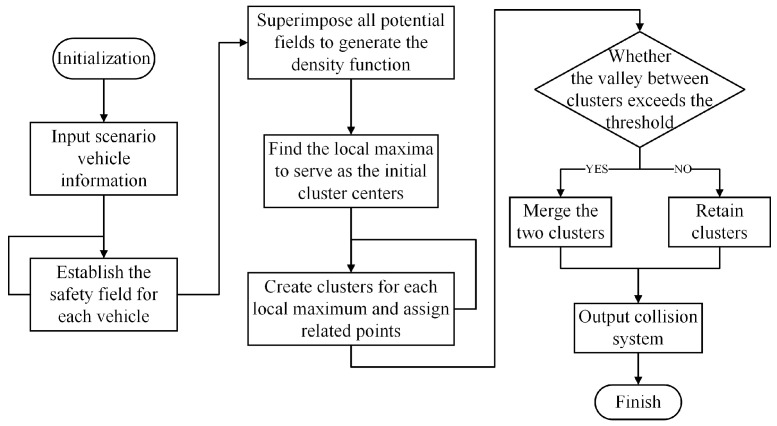
Flowchart of the DENCLUE-like method.

Imagine the entire roundabout as a data space containing a macroscopic safety field (density function). Each vehicle in the space contributes to the potential at any point in this data space. Intuitively, areas with high vehicle density should have a higher potential, while areas with fewer vehicles should have a lower potential. Thus, the construction principles of the DENCLUE algorithm are similar to those of a macroscopic safety field, making it suitable for constructing the macroscopic safety field. The flowchart of the DENCLUE-like method is shown in Figure 6.

### 3.3. Risk Degree Calculation

The risk degree of each vehicle is a key metric directly derived from its potential value. The potential value reflects the vehicle’s potential risk degree in the current environment.

Specifically, the potential value considers factors like vehicle speed, travel direction, distance to other traffic participants, and weather conditions. When a vehicle’s potential value increases, it indicates a higher risk degree, which could be due to sudden braking, unexpected obstacles, or other emergency situations. Conversely, a lower potential value indicates that the vehicle is operating in a safer state.

**Figure 7:**
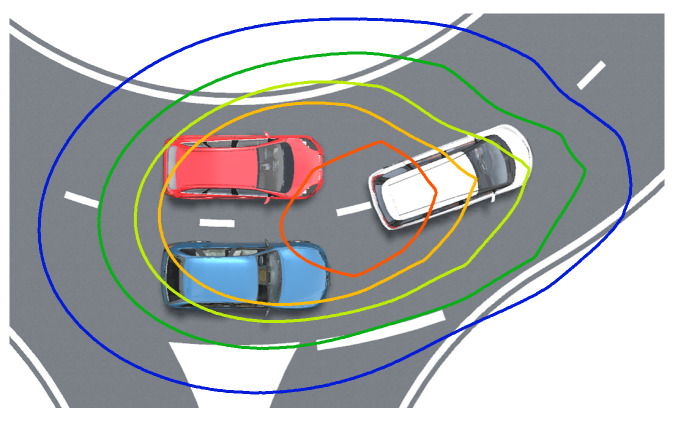
Composition of the collision system.

After DENCLUE clustering, several clusters are generated, known as collision systems. These systems exhibit high potential values, indicating heightened risk and requiring caution. Figure 7 illustrates the composition of the collision system.

According to the principles of work and energy, the process of work done by the safety field force corresponds to the migration of risk energy. When vehicles within a collision system perform negative work, it results in a further rise in risk energy. Therefore, the instability can be evaluated based on the work efficiency of the vehicles within the safety field:

(10) $$S=-\sum_{i=1}^{N}\frac{v_{i}\cos\theta_{i}}{\sum_{j=1}^{N}v_{j}},$$

where *S* is the work efficiency; *v* is the vehicle speed; θ is the angle between the safety field force and the vehicle speed; and *N* is the number of vehicles in the collision system.

The risk degree of the collision system $R_{cs}$ can be assessed through its work efficiency *S* and maximum potential value $\phi_{max}$:

(11) $$R_{cs}=\phi_{max}\cdot S.$$

The macroscopic risk degree comprehensively assesses scenario risk by focusing on the temporal distribution of potential values within the macroscopic safety field at each frame. By calculating the proportion of potential values exceeding the danger threshold at each frame, referred to as the potential value instability, and comparing it to the instability threshold of the scenario, a comprehensive risk assessment is provided. From a scenario perspective, the macroscopic risk degree has multiple advantages. It offers a global view, enabling a thorough analysis of the current scenario and helping to identify potential overall risks.

Potential value instability is determined by the following equation:

(12) $$\rho =\frac{\sum_{i=1}^{N}}{\sum_{j=1}^{M}},$$

where ρ is the potential value instability; *N* is the number of points with the potential value exceeding the safety threshold; and *M* is the number of points with potential value greater than zero.

## 4. Parameter Calibration

### 4.1. Social Force Model

The social force model [35] is based on the concept of forces and primarily consists of three components: self-driving force, boundary force, and interaction force. These forces describe the interactions and behavioral patterns between individuals. These forces are closely related to the driver characteristics and surrounding environment considered in the safety field model. Therefore, the structure of the social force model, combined with natural driving data, can be used to calibrate the parameters of the safety field model. The total effect force $F_{total}$ is defined as

(13) $$F_{total}=F_{d}+\sum F_{i}+F_{b},$$

where $F_{d}$ is the self-driving force; $F_{i}$ is the interaction force; and $F_{b}$ is the boundary force.

The self-driving force represents the driver’s desire to move from the current position toward the target position at the desired speed. If the current speed of the driver is lower than the desired speed, the self-driving force will accelerate the vehicle. The self-driving force is defined as

(14) $$F_{d}=k_{d}\cdot \left(v_{d}\cdot {\vec{e}}_{0}-\vec{v}\right),$$

where $k_{d}$ is the elasticity parameters of the self-driving force; $v_{d}$ is the desired speed parameter; ${\vec{e}}_{0}$ is the direction of the desired speed; and $\vec{v}$ is the current velocity vector.

Land et al. [36] demonstrated through experiments that drivers tend to fixate on the tangent point of the inner edge of the road ahead while navigating curves, adjusting the steering wheel angle based on the anticipated curvature to complete the turn. However, in roundabout scenarios, drivers do not consistently focus on the central island’s edge. As they approach the exit, drivers gradually shift their gaze towards it [37]. Therefore, considering the changes in drivers’ visual behavior within a roundabout, the direction of the desired speed should be tangential to the central island when entering the roundabout and gradually shift towards the exit when the vehicle is approximately 8 m away.

In the social force model, the interaction force primarily aims to avoid collisions between vehicles, ensuring a safe distance from surrounding vehicles, while the boundary force keeps the vehicle centered in the lane. The safety field force serves the same purpose as the boundary force and the interaction force in the social force model. Therefore, the safety field force can replace the boundary force and interaction force in the social force model for parameter calibration, determining the parameters in the driving safety field model. The calculation method for the safety field force is the same as that for the electric field force:

(15) $$\begin{array}{cc} F_{lane} & =E_{lane}\cdot q_{lane},\; \end{array}$$

(16) $$\begin{array}{cc} q_{lane} & =\frac{k_{l}}{d_{lane}},\; \end{array}$$

(17) $$\begin{array}{cc} F_{coll} & =E_{coll}\cdot q_{coll},\; \end{array}$$

(18) $$\begin{array}{cc} q_{coll} & =\left[\left(k_{\delta }\right)*\Delta v+k_{so}*l_{o}*w_{o}\right]*T_{o}, \end{array}$$

where $q_{lane}$ and $q_{coll}$ represent the interaction risk in the road safety field and the interactive safety field, respectively; $d_{lane}$ is the road width; Δv is the speed difference between the host vehicle and the obstacle vehicle; $l_{o}$ and $w_{o}$ are the length and width of the obstacle vehicle, respectively; $T_{o}$ is the type coefficient of the obstacle vehicles; and $k_{l}$, $k_{\delta }$ and $k_{so}$ are parameters to be determined.

### 4.2. Data Processing

The rounD dataset [38] contains public traffic data (position, speed, acceleration, and object type) recorded from a bird’s-eye view at roundabouts. It provides extensive trajectories of road users at roundabouts. The dataset was collected using drones equipped with 4K resolution cameras, capturing over 6 h of video and recording more than 13,000 road users.

The rounD dataset includes dedicated right-turn lanes, while this study focuses on vehicles traveling within the roundabout. Therefore, when extracting data for the host vehicle, the focus was on its movement within the roundabout, and relevant data from surrounding vehicles were also extracted.

In the rounD dataset, part of the south and north entrances are truncated due to the drone’s perspective. Vehicles entering from these entrances can affect the calibration process of vehicles near the entrance. Therefore, it is necessary to repair the trajectories of vehicles entering from the south and north entrances. First, the vehicle trajectories are smoothly connected to the lane centerline using Bézier curves. Then, the trajectories are resampled at 25 Hz to determine the distance changes between frames, and these distance changes are used to repair the original trajectories.

### 4.3. Parameter Calibration Method

In this study, the parameter calibration process is based on the social force model framework. First, the resultant force is calculated by integrating the improved driving safety field model into the framework. Then, according to Newton’s second law F=ma, the relationship between the resultant force and vehicle acceleration is used for parameter calibration. Specifically, we utilized acceleration data from real driving scenarios to fit and optimize the model, ensuring the accuracy and robustness of risk assessment.

As a fundamental parameter calibration method, the Least Squares Method (LSM) is renowned for its high reliability and effective solution techniques [39]. Due to these advantages, it is frequently applied in the calibration of both linear and nonlinear systems. The parameter settings for the LSM used in this study are shown in Table 4.

The error between the predicted value $Y_{pre}\left(k\right)$ and the actual value Y(k) is defined by the Root Mean Square Error (RMSE):

(19) $$RMSE=\sqrt{\frac{1}{K}\sum_{k=1}^{K}{\left(Y_{pre}\left(k\right)-Y\left(k\right)\right)}^{2}}.$$

### 4.4. Parameter Calibration Results

Based on the data processing methods described earlier, 549 vehicle driving data sets were extracted from Track 03 of the rounD dataset, and the data were repaired. To verify the accuracy of the parameters, vehicle displacement, speed, and acceleration were predicted based on the determined parameters and compared with the actual values. Taking vehicle ID 50 and vehicle ID 333 from Track 03 of the dataset as examples, these two vehicles represent straight and left-turn behaviors in the roundabout, respectively.

**Figure 8:**
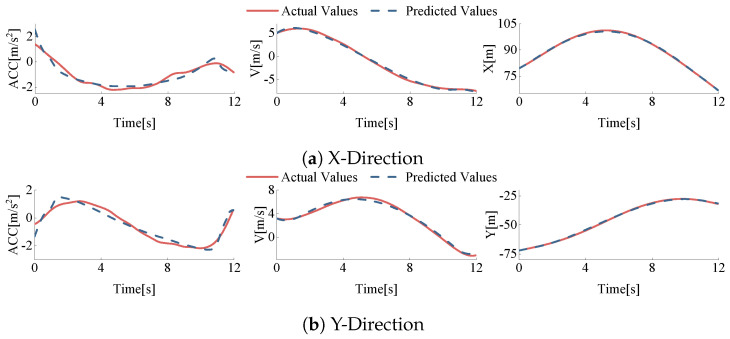
Comparison of predicted vs. actual values in X and Y directions for vehicle id50 (Track 03).

**Figure 9:**
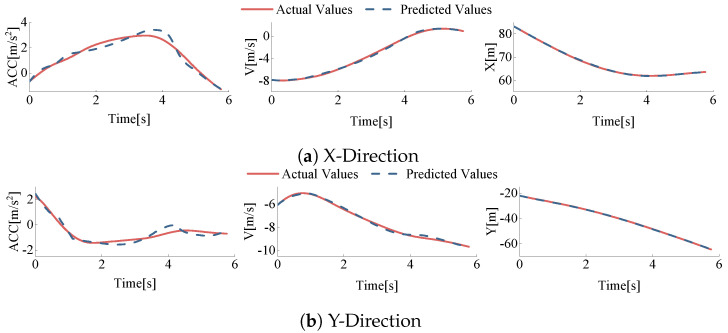
Comparison of predicted vs. actual values in X and Y directions for vehicle id333 (Track 03).

Through this analysis, we obtained the longitudinal and lateral data comparison results, as shown in Figure 8 and Figure 9. It can be seen that the errors between the predicted values and the actual values are minimal, indicating that the model parameters can accurately describe the vehicles’ driving behavior within the roundabout.

Based on the extracted roundabout driving data and calibrated parameters, the displacement, speed, and acceleration for each vehicle during lane changes were calculated. These predicted values were then compared with the actual values. The error was calculated by Equation (19), and the error values are listed in Table 5.

## 5. Simulation Analysis

To demonstrate the feasibility and effectiveness of this study, we used the playback test method to validate the proposed risk degree algorithm. The playback test method is a technique based on natural driving data, which reconstructs the trajectories of traffic participants in the real world and uses a simulation environment to recreate these real scenarios. This approach allows for precise simulation and reproduction of complex traffic scenarios, enabling a comprehensive assessment of risk degree in different situations. Specifically, this method not only realistically reproduces various traffic conditions that may be encountered during actual driving but also effectively evaluates the performance of the risk degree algorithm under different contexts, ensuring its reliability and effectiveness in real-world applications.

### 5.1. Performance Comparison

Compared to other traditional risk metrics, the advantage of Time to Collision (TTC) lies in its relatively simple calculation and wide application. Currently, collision avoidance systems or driver assistance systems in intelligent vehicles consider TTC as an important safety warning metric. The principle for selecting comparison metrics is to see whether they are suitable for the simulated scenarios and requirements of the experiment. We need to ensure that the selected risk indicators accurately reflect the actual traffic conditions and potential risks in the experiment, thereby effectively evaluating the performance and reliability of the proposed risk degree algorithm.

When it comes to the requirement for outputting instantaneous risk, indicators other than TTC have more or less deficiencies and can only highlight their advantages in specific scenarios, making comprehensive comparisons difficult. For example, Time Headway (THW) cannot evaluate the safety of vehicles during lane changes or overtaking, and Post-Encroachment Time (PET) cannot represent the risk degree at a fixed point in time, making it unsuitable for comparisons since the focus of this paper is on the changes in safety performance at each moment.

TTC is typically used in straight-following scenarios, but in roundabout scenarios, directly using relative distance divided by relative speed is clearly inappropriate. Therefore, the TTC calculation method needs to be modified to be as effective as possible in roundabout scenarios [40]. TTC in roundabout scenarios can be calculated as fololows:

(20) $$TTC=\frac{R\cdot \theta }{v_{host}-v_{obstacle}},$$

where $v_{host}$ is the speed of the host vehicle; $v_{obstacle}$ is the speed of the other vehicle; θ is the angle between the lines connecting the centers of the two vehicles to the roundabout center; and *R* is the average radius of the circular movement of the two vehicles around the roundabout center.

Due to the fact that TTC can have negative and infinite values, resulting in a wide range of data, it is scaled by taking the reciprocal of TTC to obtain TTCi. The larger the TTCi, the more dangerous the vehicle is. According to the review by Li et al. [16], 1.5 s of TTC is commonly used as a conflict threshold in roundabout scenarios, which corresponds to a TTCi conflict threshold of 0.66.

### 5.2. Case 1

Merging scenarios frequently occur in roundabouts, as almost every vehicle entering the roundabout must perform one or more merging maneuvers. The high frequency of these merging actions makes studying their safety of great practical significance. Merging in roundabouts involves complex interactions between multiple lanes and vehicles, requiring drivers to make several decisions and maneuvers in a short period, significantly increasing the complexity of the merging process. Although the primary design of roundabouts aims to improve traffic efficiency and safety, the merging areas within roundabouts remain hotspots for traffic accidents due to driver unfamiliarity and complex traffic conditions.

Accurately assessing the risk of merging behavior is crucial for evaluating vehicle driving risk in roundabout scenarios. By precisely analyzing the risk factors and interaction complexity of merging behavior, important foundations can be provided for defining risk degree in roundabout scenarios. In roundabouts, the merging behavior of vehicles involves complex interactions between multiple lanes and vehicles, which can easily lead to a series of traffic risks. These risks include misjudgments caused by obstructed views, rear-end or side collisions due to speed differences, and the uncertainty of driver decision-making behavior. These factors collectively increase the accident rate in merging scenarios within roundabouts.

**Figure 10:**
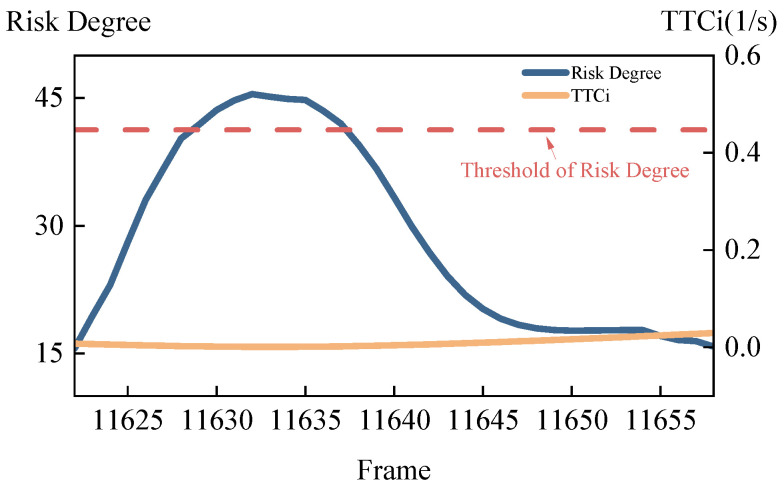
Comparison of the host vehicle’s risk degree and TTCi in scenario 1.

Scenario 1 starts at frame 11,622 and ends at frame 11,658, with a duration of 1.44 s. The comparison of the host vehicle’s risk degree and TTCi in scenario 1 is shown in Figure 10. It can be observed that the risk degree exceeds the danger threshold between frames 11,629 and 11,637, whereas TTCi remains below the danger threshold throughout the entire process.

**Figure 11:**
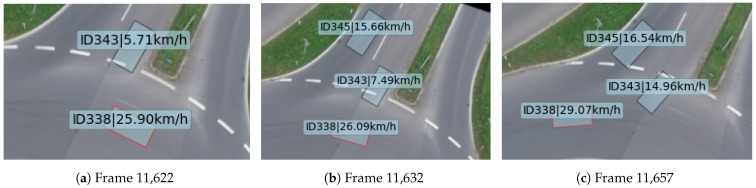
The schematic of scenario 1.

At frame 11,622 (as shown in Figure 11a), the host vehicle (id338) is about to pass through the roundabout intersection. At this moment, there is a car, Car A (id343), within the intersection, traveling at a speed of 5.71 km/h and entering the roundabout. The host vehicle is traveling at a speed of 25.90 km/h.

At frame 11,629, the host vehicle’s risk degree exceeds the threshold. At this moment, Car A is directly beside the host vehicle, with its speed increased to 6.61 km/h and the distance to the host vehicle decreased, while it continues to accelerate. The risk degree peaks at frame 11,632 (as shown in Figure 11b), at which point there are two cars within the roundabout intersection: Car A, traveling at 7.49 km/h, and Car B (id345), traveling at 15.66 km/h. However, Car B is farther from the host vehicle and poses less risk.

After frame 11,632, as Car A gradually completes its merging behavior, the risk shifts from the side of the host vehicle to the rear. Consequently, the risk degree gradually decreases, falling below the threshold after frame 11,637, allowing the host vehicle to continue circulating within the roundabout (as shown in Figure 11c).

However, throughout the entire simulation process of scenario 1, TTCi consistently remained below the danger threshold, indicating that TTCi failed to detect the risk during the simulation of scenario 1. In contrast, the proposed safety model accurately assessed the risk in the merging scenario.

### 5.3. Case 2

The traffic flow characteristics of roundabouts make the evaluation of multi-vehicle conflicts particularly important. The primary design intention of roundabouts is to reduce wait times and dependence on traffic signals through continuous traffic flow, but this design also results in more complex traffic interactions. In roundabouts, vehicles must not only manage risks from the front and rear but also closely monitor other vehicles entering, exiting, and operating within the roundabout from all directions. This multi-directional traffic flow increases the potential conflict points, making it crucial to assess conflicts in each direction and between every vehicle.

**Figure 12:**
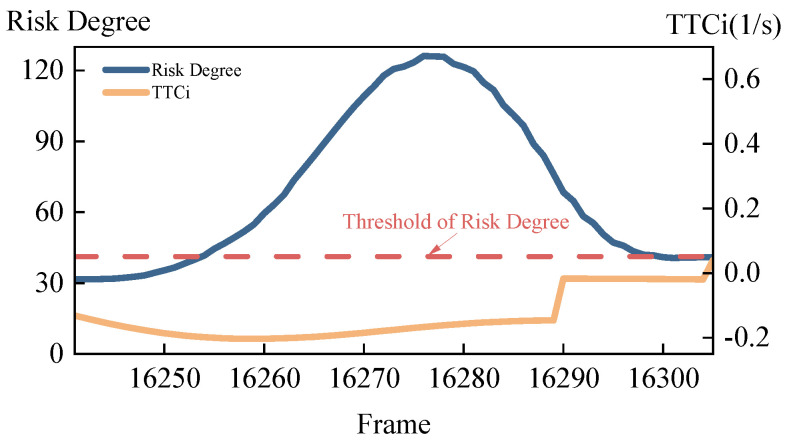
Comparison of the host vehicle’s risk degree and TTCi in scenario 2.

Scenario 2 starts at frame 16,241 and ends at frame 16,305, with a duration of 2.56 s. The comparison of the host vehicle’s risk degree and TTCi in scenario 2 is shown in Figure 12. It can be observed that the risk degree exceeds the danger threshold between frames 16,254 and 16,299, whereas TTCi remains below the danger threshold throughout the entire process.

**Figure 13:**
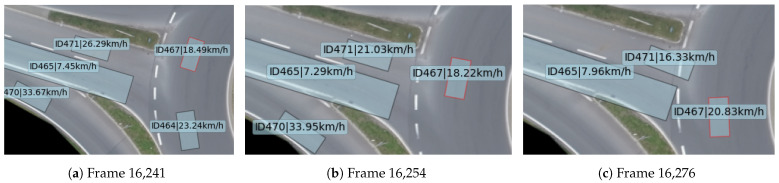
The schematic of scenario 2.

At frame 16,241 (as shown in Figure 13a), the host vehicle (id467) is about to pass through the roundabout intersection. At this moment, there are two vehicles within the intersection: Truck C (id465) and Car D (id471), traveling at speeds of 7.45 km/h and 26.29 km/h, respectively. The host vehicle is traveling at a speed of 18.49 km/h. Truck C and Car D are about to enter the roundabout, resulting in a trajectory conflict with the host vehicle, specifically a merging conflict.

Subsequently, the host vehicle’s risk degree continues to rise, exceeding the threshold at frame 16,254 (as shown in Figure 13b), indicating that the host vehicle is in a dangerous state. At this point, the host vehicle is passing through the intersection, with Truck C and Car D traveling at speeds of 21.03 km/h and 7.29 km/h, respectively. Compared to frame 16,241, the distance between Truck C and Car D and the host vehicle gradually decreases.

The risk degree reaches its peak at frame 16,276 (as shown in Figure 13c), at which point the host vehicle has passed through the first lane of the intersection and is traveling at a speed of 20.83 km/h. Car D has entered the roundabout and is moving at a speed of 16.33 km/h. Truck C is located to the side of the host vehicle, moving at a speed of 7.96 km/h. The host vehicle’s risk degree increases to its highest point in the entire scenario due to the influence of the safety fields from Truck C, which is merging from the side, and Car D, which is merging from behind. After frame 16,276, Truck C and Car D gradually complete their merging behaviors, and the host vehicle exits the intersection. Consequently, the risk degree gradually decreases and falls below the threshold by frame 16,300.

Throughout the entire process of scenario 2, the TTCi remains consistently below the danger threshold of 0.66, indicating that TTCi fails to detect the risk during the simulation of scenario 2. Therefore, the proposed risk calculation method not only accurately assesses omnidirectional risks but also precisely evaluates the risks associated with merging behaviors and multi-vehicle conflicts.

**Figure 14:**
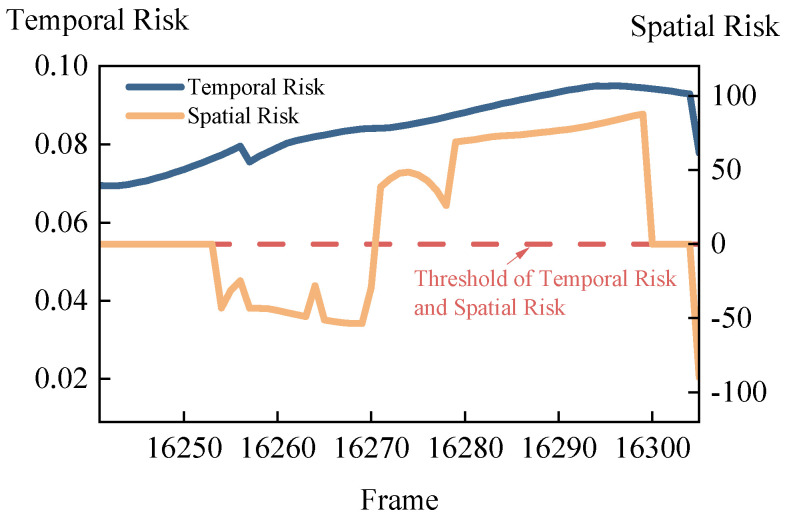
Comparison of temporal risk and spatial risk.

As previously mentioned, the temporal risk of a scenario is represented by the macroscopic risk degree. The spatial risk of a scenario is represented by the collision system risk degree. The collision system is formed by interacting vehicles. By assessing the risk degree of the collision system in which the host vehicle is located, the host vehicle and its interacting vehicles can be evaluated as a whole to determine if the danger occurs at the host vehicle’s location. Comparing spatial risk degree with temporal risk degree allows for the elimination of scenarios without a testing value, thereby improving the efficiency and accuracy of virtual testing. The comparison of temporal risk and spatial risk is shown in Figure 14.

It can be observed that the temporal risk remains above the threshold throughout the entire simulation, indicating a high proportion of high-risk within the scenario, making the scenario quite dangerous. The spatial risk exceeds the threshold between frames 16,271 and 16,299, indicating that during this period, the danger is concentrated within the collision system formed by the host vehicle and its interacting vehicles. By comparing temporal risk and spatial risk, we can explore the test value of dangerous scenarios and provide guidance for virtual testing.

Simulations were conducted on Track 03 in rounD to compare the performance of the TTC method with the proposed method. The results show that the TTC method detected 28 dangerous scenarios, all related to the driving direction of the vehicle. In contrast, the proposed method detected a total of 77 dangerous scenarios, including 28 driving direction risks, 26 multi-vehicle conflicts, and 23 omnidirectional risks. Compared to the TTC method, the proposed method significantly improves the efficiency of detecting dangerous scenarios, with the number of detected dangerous scenarios increasing by approximately 175%.

This significant improvement indicates that the proposed method offers notable advantages in detection efficiency and accuracy in complex roundabout scenarios where traditional methods may struggle. The model’s ability to capture a wider range of hazardous situations, including multi-vehicle conflicts and omnidirectional risks, enables a more comprehensive and precise quantification of driving risks. Such enhanced detection capabilities not only prove valuable in simulation environments but also underscore the practical potential for real-world applications. When integrated into autonomous vehicle systems, this model can significantly enhance real-time risk identification and decision-making processes. By accurately identifying diverse and complex hazards, it can help vehicles navigate challenging environments like roundabouts more safely, reducing the likelihood of accidents caused by multi-directional conflicts and unexpected interactions with other vehicles.

Moreover, in scenario-based testing, the proposed method enables the more efficient identification and localization of hazardous scenarios, significantly improving testing efficiency. This enhancement not only reduces the time needed to find high-risk scenarios but also provides more comprehensive coverage of potential risk scenarios, making the testing process both more efficient and precise. This is particularly valuable in virtual testing, as it allows for the generation of more representative hazardous scenarios in a shorter time frame, thereby further enhancing the validation of autonomous driving systems’ safety.

## 6. Conclusions

This paper proposes an improved safety field model based on field theory that considers traffic flow characteristics at roundabouts. The model accurately assesses risks associated with vehicle diverging and merging in roundabouts. It unifies the models of dynamic and static traffic participants and dynamically characterizes the spatial distribution of safety risks around vehicles in different motion states. Subsequently, the parameters are calibrated using the social force model based on the entire driving process data. Finally, a DENCLUE-like method is used to extract the collision systems formed by interacting vehicles, enabling the evaluation of individual vehicle and scenario risks.

Comparative results show that the proposed method more accurately reflects interaction risks at roundabout intersections, assessing omnidirectional risks and multi-vehicle conflicts more effectively, making it more suitable for roundabout scenarios. Additionally, comparing temporal and spatial risks helps guide critical scenario generation, improving virtual testing efficiency.

The model presented in this study has limitations regarding its transferability. Specifically, its effectiveness in diverse traffic environments has not yet been established. This research and its experiments concentrate on roundabout scenarios, chosen for their inherent complexity and distinct vehicle interactions. The modeling and analysis were tailored to these specific conditions. Future work will aim to adapt the model for other complex traffic environments. For example, in signal-controlled intersections, it will be crucial to account for the effects of traffic lights on vehicles and re-model these constraints using a force-based approach. Additionally, refining the model to accommodate the unique characteristics of different scenarios will be important. Nonetheless, the risk assessment framework developed in this study can still provide valuable insights for research on other complex scenarios.

In summary, this research advances intelligent connected vehicle safety by addressing the unique challenges of roundabout scenarios and offering a robust risk assessment framework. Future studies should continue to enhance the model’s transferability and explore its integration into broader traffic management systems.

## Figures and Tables

**Table 1:** Type coefficient results.

Type Coefficient	Value
$T_{car}$	1.0
$T_{van}$	1.0454
$T_{truck}$	1.4077

**Table 2:** Weather coefficients for different weather conditions.

Weather Conditions	Value
No precipitation	0.9
Rain	1.06
Sleet	1.46
Snow	2.18

**Table 3:** Coefficient values for road potential field.

Coefficient	Value
$k_{LT}$ for the lane line	2
$k_{LT}$ for the boundary line	8
Shape coefficient *n*	3

**Table 4:** LSM parameter settings.

LSM Options	Value
FunctionTolerance	$10^{-6}$
StepTolerance	$10^{-6}$
MaxFunctionEvaluations	2000
MaxIterations	1000

**Table 5:** RMSE between predicted and actual values.

	Disp (m)	V (m/s)	Acc (m/s^2^)
X-dir	0.5514	0.4791	0.5858
Y-dir	0.6339	0.5261	0.6018

## Data Availability

The original data presented in the study are openly available at https://levelxdata.com/round-dataset/ (accessed on 3 January 2024).
